# Assessment of Serum Resistin and Plasma Calprotectin Levels as Biomarkers of Inflammation in Patients with Familial Mediterranean Fever Disease

**DOI:** 10.31138/mjr.33.3.322

**Published:** 2022-09-30

**Authors:** Emrah Kılıçaslan, Tolga Düzenli, Serkan Çelik, Mustafa Kaplan, Çağatay Öktenli, Cihan Top

**Affiliations:** 1 Department of Hematology, Göztepe Prof. Dr. Süleyman Yalçın City Hospital, Istanbul, Turkey,; 2 Department of Gastroenterology, SBÜ Sancaktepe Şehit Prof. Dr. İlhan Varank Training and Research Hospital, Istanbul, Turkey,; 3 Department of Medical Oncology, BAU Medicine School Göztepe Medical Park Hospital, Istanbul, Turkey,; 4 Department of Internal Medicine, Sultan 2 Abdulhamid Han Training and Research Hospital, Istanbul, Turkey,; 5 Department of Internal Medicine, Anadolu Medical Center, Kocaeli, Turkey,; 6 Department of Internal Medicine, Florence Nightingale Hospital, Istanbul, Turkey

**Keywords:** familial Mediterranean fever, resistin, calprotectin, inflammation

## Abstract

**Objective:**

While several inflammatory markers are known to increase in familial Mediterranean fever (FMF) disease cases, the need remains for diagnostic tests specific for FMF that monitor inflammatory activity. We aimed to investigate resistin and calprotectin levels during both attack and attack-free periods of FMF disease and evaluate their use as novel biomarkers of inflammation in patients with FMF.

**Materials and Methods:**

This cross-sectional study included 68 male patients diagnosed with FMF and 20 healthy individuals as controls. Blood samples were obtained from the patients in attack-free periods (at least 15 days after the last attack) and attack periods (in the first 24 hours). Serum resistin and plasma calprotectin levels was measured by ELISA method.

**Results:**

Resistin and calprotectin levels were significantly higher in patients during both attack (p =0.001, p <0.001) and attack-free periods (p =0.017, p =0.01) compared to the control group. Logistic regression analysis indicated that resistin levels were predictive for the diagnosis of FMF disease (OR: 1.21; 95% CI: 1.04–1.42; p =0.016). Resistin and calprotectin levels significantly correlated with C-reactive protein, erythrocyte sedimentation rate, fibrinogen, and white blood cells (0.301≤ r ≤ 0.505, p <0.05).

**Conclusion:**

Resistin and calprotectin levels were significantly higher in patients than controls, and resistin was predictive for monitoring inflammatory activity in patients with FMF.

## INTRODUCTION

Familial Mediterranean fever (FMF) is a genetic disease resulting from Mediterranean FeVer (MEFV) gene variations.^2,3^ This MEFV gene encodes the protein of pyrin, which plays an important role in inflammation and apoptosis.^2–4^ The autoinflammatory disease FMF, characterized by short-lived recurrent episodes of peritonitis, pleuritis, arthritis, rash, and fever^1^, is commonly detected in Sephardic Jews, Armenians, Turks, Greeks, Arabs, and Italians in the Mediterranean region.^1,2^ A diagnosis of FMF is currently based on clinical findings according to the Tel Hashomer and recently published Eurofever/PRINTO classification criteria which also includes genetic analysis.^1–5^ While currently there is no definitive diagnosis for FMF, ethnicity, family history, and mutation in the MEFV gene support the diagnosis in patients with clinical findings. Other findings that contribute to an FMF diagnosis are high blood levels of acute-phase reactants (APR) during attack periods. The major mechanisms of pathogenesis for clinical manifestations include the over-activation of cytokine cascades.^3^ Abnormal pyrin protein, which is proposed to result from MEFV gene mutations, is suggested to precipitate ineffective suppression of inflammation responsible for the inflammatory process.^3^ Resistin is a regulatory cytokine that triggers the pro-inflammatory state by increasing the synthesis of cytokines such as TNF-α, IL-1β, and IL-6. Calprotectin is a heterodimer belonging to the family of S-100 calcium-binding proteins and has been studied as an inflammatory indicator in several diseases, including FMF.

Difficulties lead to delays in diagnosis of FMF, with the main problems being atypical clinical presentations that do not fully meet the diagnostic criteria and overlap diseases.^6^ Considering that the average delay in diagnosis of FMF is 7.3 years, one can speculate that new, reliable, faster parameters may be useful for a more expedient diagnosis.^7^ In this study, we aimed to evaluate serum resistin and plasma calprotectin in patients during both attack and attack-free periods of FMF and investigate the role of serum resistin and plasma calprotectin levels in the diagnosis and activity of FMF disease.

## MATERIALS AND METHODS

### Selection of study groups

A total of 68 male patients diagnosed with FMF and 20 healthy age-matched males were enrolled in this cross-sectional study. The local ethics committee of GATA Haydarpaşa Training and Research Hospital approved the study with a decision number of 157/2010. Written informed consent was obtained from all participants.

The FMF attack period was diagnosed by Tel Hashomer criteria and combined high APR levels with positive physical examinations. In terms of phenotype characteristics, all patients had peritonitis.

Patient findings were recorded for further evaluation and classification. Blood samples were obtained during attack-free periods (at least 15 days after the last attack) and attack periods (in the first 24 hours).

Patients with a body mass index > 30 kg/m^2^, using non-steroidal anti-inflammatory drugs, steroids, immunosuppressants, or immune regulatory drugs, with impaired cognitive function, cardiovascular disease, acute or chronic liver or kidney disease, chronic obstructive pulmonary disease, rheumatological disease, malignancy, thyroid disease, immune deficiency, hypertension, diabetes mellitus, acute or chronic infection, bleeding diseases, and those who did not meet the study criteria were excluded. All patients diagnosed previously with FMF were using 1–1.5 mg/day of colchicine. Patients who did not use colchicine regularly were excluded from the study. Patients were routinely monitored for liver enzymes, complete cell blood count, kidney functions, creatinine phosphokinase (CPK) and proteinuria for amyloidosis every six months and patients with amyloidosis were excluded from the study.

### Study protocol and tests

Peripheral venous blood samples were taken from FMF patients during an attack-free period at least 15 days from the last attack and following 8-hour fasting and within 24 hours after an acute attack onset to assess complete blood count (CBC), erythrocyte sedimentation rate (ESH), C-reactive protein (CRP), and fibrinogen. Serum and plasma, obtained by centrifuging 5-ml blood samples at 5000 g for 5 minutes, were stored at −80°C in 1ml Eppendorf tubes until tested in the biochemistry laboratory.

Serum resistin levels (AssayPro Human Resistin ELISA Kit, Cat. No. ER1001-1, Lot No. 03571107, CIOM-China device) and plasma calprotectin levels (Hycult Biotech Human Calprotectin ELISA Kit, Cat. No. HK325, Lot No. 10003K0610-A, CIOM-China device) were evaluated by ELISA according to the kit manufacturer’s recommendations.

### Statistical analysis

The SPSS software program version 20.0 (SPSS Inc, Chicago, IL, USA) was used for statistical analysis. Continuous variables were examined with a Kolmogorov-Smirnov test for normal distribution. Differences between more than two groups were evaluated by variance analysis (one-way ANOVA). The differences between two groups were examined with the Mann-Whitney U test or Student’s t-test. Pearson and Spearman correlation tests were used for correlation analysis. The efficiency of the variables for the diagnosis of FMF was evaluated by logistic regression analysis. A P <0.05 was considered statistically significant.

## RESULTS

Demographic and clinical data of patient groups are presented in **Table 1**. The data in table 1 was presented as median (Min-Max). Resistin and calprotectin levels were significantly higher in patients in both attack (p <0.001, p =0.007) and attack-free periods (p <0.001, p <0.001) than in the control group. Calprotectin levels were higher in attack periods than attack-free periods (p =0.004). There was no significant difference between attack and attack-free period resistin levels (p =0.122).

**Table 1.** Demographic characteristics and laboratory results of the groups.

	**Patients with attack (n =34)**	**Patients attack-free (n =34)**	**Control group (n =20)**	**p^a^**	**p^b^**	**p^c^**
Age (years)	22 (20–37)	21 (18–31)	20 (20–29)	0.245	0.581	0.112
BMI (kg/m^2^)	23.1 (17.8–29.4)	23.9 (19.3–27.8)	23.5 (20.9–28.1)	0.764	1	0.795
WBC (x10^3^/μl )	10.3 (5.3–14.6)	7.0 (5.1–10.3)	5.8 (4.8–10)	<0.001 *	0.061	<0.001 *
Fibrinogen (mg/dl)	563 (421–898)	334 (5–631)	258 (210–417)	<0.001 *	0.003 *	<0.001 *
ESR (mm/h)	46 (2–101)	11 (2–78)	6 (2–24)	<0.001 *	0.02 *	<0.001 *
CRP (mg/l)	96 (22–204)	11 (2–72)	3 (1–12)	<0.001 *	<0.001 *	<0.001 *
Resistin (ng/ml)	30.9 (9–149.1)	23.3 (8.7–144.7)	9.3 (3.5–22.1)	0.122	<0.001 *	<0.001 *
Calprotectin (ng/ml)	940.5 (633.2–1345.3)	866.4 (173.1–1099.1)	588.1 (161.7–1166.5)	0.004 *	0.007 *	<0.001 *
Disease duration (year)	14.5 (2–23)	15 (2–25)				

BMI: body mass index; WBC: white blood cells; ESR: erythrocyte sedimentation rate; CRP: C-reactive protein.

* p <0.05 was considered statistically significant.

a Comparison of attack and attack-free FMF patients

b Comparison of attack and control groups

c Comparison of attack-free FMF patients and control group

**Figure 1.**
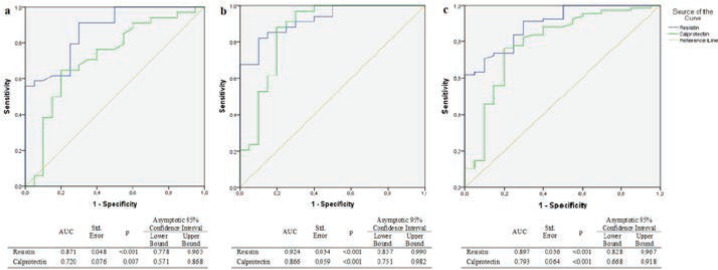
Receiver operating characteristic analysis of resistin and calprotectin for FMF patients and healthy individuals: (**a**) ROC curve of attack-free period and control group; (**b**) ROC curve of attack period and control group; (**c**) ROC curve of all FMF patients (attack and attack-free periods) and control group.

Receiver operating characteristic (ROC) curve analysis revealed a cut-off resistin value of 11.92 ng/ml with 91.2% sensitivity and 70% specificity (p <0.001) and for calprotectin as 391.44 ng/ml with 95.6% sensitivity and 40% specificity (p <0.001) (**Table 2**, **Figure 1**).

**Table 2.** Receiver operating characteristic analysis of resistin and calprotectin in FMF patients.

	**AUC (95%)**	**Cut-off value**	**p**	**% Sensitivity**	**% Specificity**
Resistin (ng/ml)	0.897 (0.828–0.967)	11.92 ng/ml	<0.001 *	91	70
Calprotectin (ng/ml)	0.793 (0.668–0.918)	391.44 ng/ml	<0.001 *	96	40

AUC: area under the curve; ng/ml: nanogram/millilitre

* p <0.05 was considered statistically significant.

Logistic regression analysis indicated that resistin levels were predictive for the diagnosis of FMF disease in both attack and attack-free periods (OR: 1.21; 95% CI: 1.04–1.42; p =0.016) **(Table 3)**, while calprotectin had no significant predictive role in attack and attack-free periods (p =0.547). Resistin and calprotectin levels significantly correlated with CRP, ESR, fibrinogen, and white blood cells (0.376≤ r ≤ 0.522, p <0.001) (**Table 4**).

**Table 3.** Logistic regression analysis of parameters associated with FMF.

	**p**	**Odds ratio**	**95% CI for EXP(B)**	
			**Lower**	**Upper**
WBC	0.683	1	1	1.001
Fibrinogen	0.915	1.001	0.988	1.014
ESR	0.734	1.023	0.895	1.17
CRP	0.147	1.184	0.943	1.487
Resistin	0.016 *	1.213	1.036	1.42
Calprotectin	0.547	0.999	0.996	1.002

WBC: white blood cells; ESR: erythrocyte sedimentation rate; CRP: C-reactive protein.

* p <0.05 was considered statistically significant.

**Table 4.** Correlation analysis of resistin and calprotectin with inflammatory parameters in FMF disease.

	**Age**	**BMI**	**CRP**	**ESR**	**Fibrinogen**	**WBC**	**Calprotectin**
**Resistin**	r=− 0.011	r=−0.218	r=0.519	r=0.417	r=0.454	r=0.376	r=0.497
	p=0.921	p=0.041 *	p=<0.001 *	p=<0.001 *	p=<0.001 *	p=<0.001 *	p=<0.001 *
**Calprotectin**	r=0.154	r=−0.118	r=0.522	r=0.424	r=0.512	r=0.390	
	p=0.152	p=0.274	p<0.001 *	p<0.001 *	p<0.001 *	p<0.001 *	

BMI: body-mass index; CRP: C-reactive protein; ESR: erythrocyte sedimentation rate; WBC: white blood cells.

* p <0.05 was considered statistically significant.

## DISCUSSION

The current study revealed that resistin and calprotectin levels were significantly higher in patients during attack and attack-free periods than in a control group. Calprotectin was also found to be useful in differentiating between attack and attack-free periods of FMF.

Higher ESR, CRP, fibrinogen, white blood cell count, and serum amyloid A (SAA) are expected results in active FMF disease compared to the attack-free period.^8,9^ However, in a systematic review investigating APR used for FMF diagnosis, Erer et al. reported that there was no effective APR to diagnose FMF disease.^9^

The innate immune system in FMF patients is proposed to be disrupted, and the disease progresses with episodes of systemic inflammation.^10^ The innate immune system activates the adaptive immune system by antigen-presenting cells. Thus, B and T cells respond and result as disease symptoms.^10^ Cytokines IL-1β, IL-1α, TNF α, TNF β, and IL-6 play an important role in these mechanisms.^5^ Also, IL-1β and NF-κB pathways are abnormally activated due to mutation in the C terminal B30.2 region of pyrin.^11^ Resistin and calprotectin are effective over NF-κB, which plays a role in the centre of inflammation. In light of these mechanisms, our findings suggest that high resistin and calprotectin levels can be relevant diagnostic markers for FMF disease.

Resistin is a regulatory cytokine that triggers the pro-inflammatory state by increasing the synthesis of cytokines such as TNF-α, IL-1β, and IL-6.^12^ Resistin targets toll-like receptor 4 (TLR4) or adenylyl cyclase-associated protein 1 (CAP1). Upon binding to TLR4 and CAP1, resistin can trigger various intracellular signal transduction pathways to induce inflammation.^13^ Resistin is synthesised in bone marrow, trophoblastic cells, the pancreas, leukaemia cells, synovial tissue, adipose tissue, and mostly in human peripheral blood mononuclear cells. The serum resistin level increases in some inflammatory diseases. Rheumatoid arthritis has a strong correlation with serum resistin levels.^14^ Kisacik et al. reported that resistin levels could be useful in diagnosing FMF patients with attacks, but it was not useful in the differential diagnosis of acute appendicitis.^15^ In the present study, resistin studied in both attack and attack-free situations was significantly higher in FMF patients than in healthy individuals and was predictive of disease according to logistic regression analysis.

Calprotectin, a heterodimer belonging to the family of S-100 calcium-binding proteins, has been studied as an inflammatory indicator in several diseases, including FMF. Asan et al. compared attack-free FMF patients with healthy individuals and indicated that serum calprotectin levels were significantly higher in attack-free FMF patients than in healthy controls.^16^ Altug Gucenmez et al. also noticed in a paediatric age group that faecal calprotectin levels of attack-free FMF patients were higher than those of healthy controls.^17^ The authors suggested that there might be a subclinical intestinal inflammation related to autoinflammatory processes. Demirbas et al. evaluated faecal calprotectin in children with FMF during the non-attack period and also reported that faecal calprotectin was higher in FMF patients than in healthy children.^18^ However, plasma calprotectin in patients with attacks has not yet been studied. We believe that our findings have taken steps forward by evaluating FMF patients during both attack and attack-free periods.

In our study, logistic regression analysis indicated that high resistin levels were predictive for FMF disease. We think that the major advantage of resistin over common widely available conventional APR would be its usefulness for predicting both attack and attack-free FMF patients, while conventional APR is generally used in attack periods. In addition, ROC analysis revealed satisfactory sensitivity and specificity scores for resistin and calprotectin. There is a need for further prospective studies to evaluate resistin and calprotectin in different periods of FMF disease and their role in the differential diagnosis.

An interesting result of our study was that calprotectin but not resistin was higher in attack periods than attack-free periods of FMF patients. This result may indicate that calprotectin is especially effective during attack period pathogenesis, or vice versa; resistin is more effective for identifying subclinical inflammation in attack-free periods. It was previously shown that neutrophil extracellular traps (NETs) play an important role in the pathogenesis of FMF.^19–21^ During FMF attack, neutrophils release chromatin structures called NETs, which are decorated with bioactive IL-1β.^19^ The calprotectin as a neutrophil related protein maybe one of the NET-associated proteins and quantitative analysis of these proteins reflect to NET formation and disease exacerbation. Logistic regression analysis, including both attack and attack-free samples, also supported the finding that resistin might be more effective for showing subclinical inflammation in attack-free periods. Subclinical inflammation is an insidious feature of FMF, and it is not known exactly whether this persistent inflammation is associated with any particular phenotype. Babaoglu et al. analysed the predictors of persistent subclinical inflammation in a comprehensive study and demonstrated the risk factors for persistent inflammation as male gender, history of exertional leg pain, inflammatory comorbidities, M694V homozygosity, colchicine resistance, and musculoskeletal attack dominance.^22^ In this regard, we believe that future studies examining patients who have these risk factors would be valuable to evaluate the effectiveness of resistin, calprotectin, and other novel biomarkers.

Another finding in our study was that resistin and calprotectin correlated with CRP, ESR, WBC, and fibrinogen. This correlation consolidates the potential of resistin and calprotectin as effective inflammatory biomarkers.

There are other new candidate biomarkers for FMF in the literature.^8,23–28^ Pentraxin-3, omentin, serum CXC chemokine ligand 16, serum amyloid A, CD144^+^ and CD146^+^ as circulating endothelial microparticles, chitotriosidase, S10012A, and resolvin D1 have been investigated and had promising results. However, there is still a need for prospective large cohort studies before using these novel biomarkers in daily practice.

This study has some limitations. First, the study population was small, and second, all study participants were male because the study was conducted at a tertiary military centre. The colchicine treatment may influence the results, as it has anti-inflammatory effects and can cause a decrease in pro-inflammatory cytokine levels.^2–4^ Also, due to the cross-sectional design of our study, the samples reflect only one time period.

We expect that these limitations do not weaken our results due to the efficient homogenization between the groups. In addition, our study population was young with no confounding co-morbid situations. Thus, our strict exclusion criteria render our research valuable.

In conclusion, we showed that resistin and calprotectin levels were significantly higher in patients with FMF than in healthy individuals. Further prospective, randomised, large studies are needed to elucidate the roles of resistin and calprotectin in the pathogenesis of FMF.
